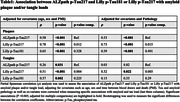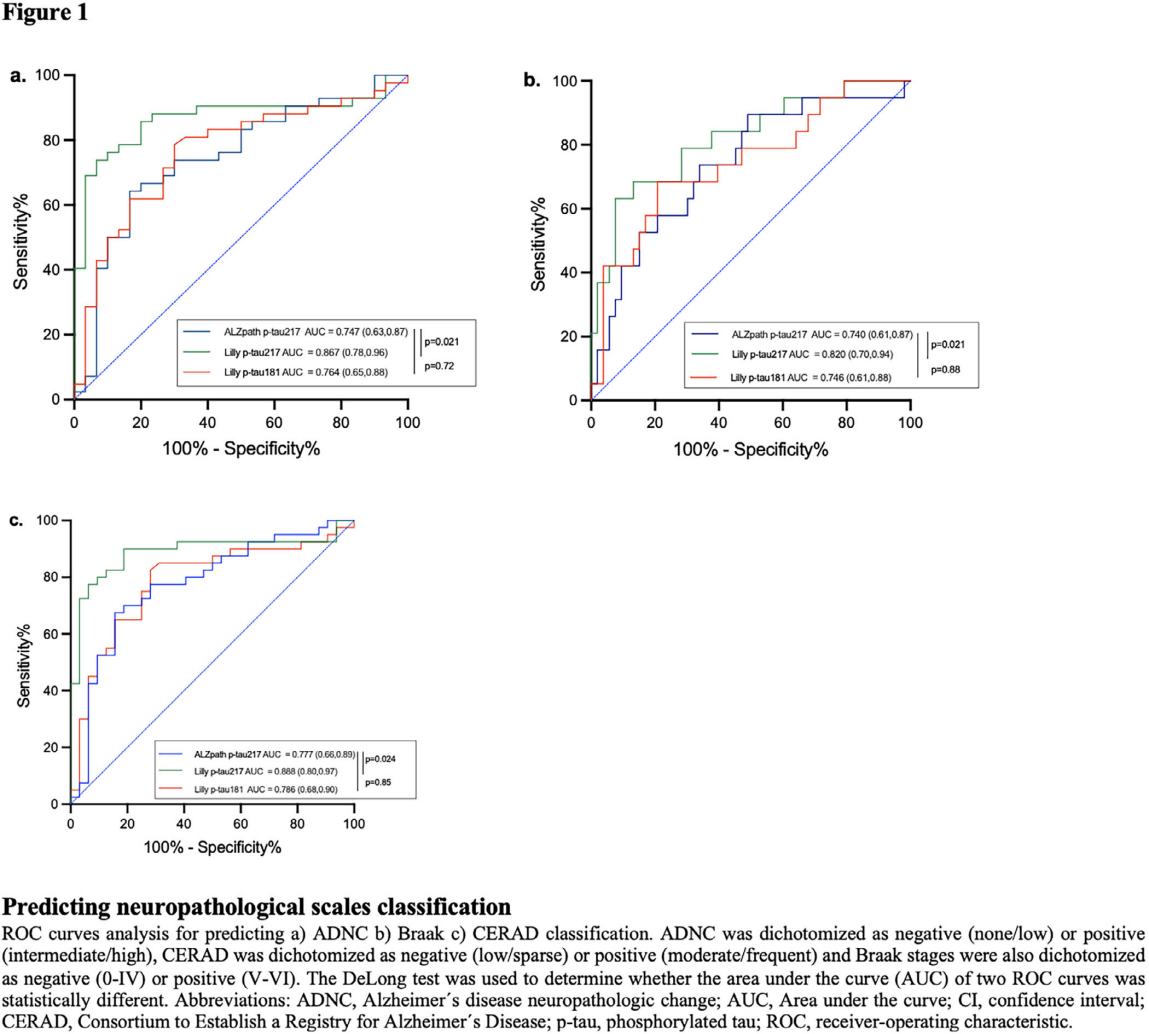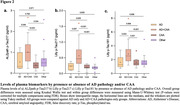# Comparison of plasma ALZpath p‐Tau217 with Lilly p‐Tau217 and p‐Tau181

**DOI:** 10.1002/alz.088984

**Published:** 2025-01-09

**Authors:** Divya Bali, Shorena Janelidze, Gemma Salvadó, Geidy E Serrano, Thomas G. Beach, Andreas Jeromin, Oskar Hansson

**Affiliations:** ^1^ Clinical Memory Research Unit, Department of Clinical Sciences, Lund University, Lund, Skåne Sweden; ^2^ Clinical Memory Research Unit, Department of Clinical Sciences, Lund University, Malmö Sweden; ^3^ Clinical Memory Research Unit, Department of Clinical Sciences, Lund University, Lund Sweden; ^4^ Civin Laboratory for Neuropathology, Banner Sun Health Research Institute, 10515 W Santa Fe Drive, Sun City, AZ 85351, Sun City, AZ USA; ^5^ ALZpath. Inc, Carlsbad, CA USA; ^6^ Clinical Memory Research Unit, Department of Clinical Sciences Malmö, Faculty of Medicine, Lund University, Lund Sweden; ^7^ Skåne University Hospital, Malmö, 21428 Skåne Sweden

## Abstract

**Background:**

There is an urgent need for accurate and validated methods to measure plasma phosphorylated tau (p‐Tau) biomarkers in Alzheimer´s disease (AD). Here we compared the performance of newly developed plasma ALZpath p‐Tau217 assay to other established p‐Tau assays such as Lilly p‐Tau217 and Lilly p‐Tau181.

**Method:**

We included 72 participants from the Arizona Study of Aging and Neurodegenerative Disorders cohort, where we analysed antemortem collected plasma samples with ALZpath p‐Tau217 as well as Lilly p‐Tau217 and p‐Tau181. The plasma biomarkers were compared with relevant post‐mortem neuropathological measures.

**Result:**

We found that ALZpath p‐Tau217 correlated significantly with Lilly p‐Tau217 and Lilly p‐Tau181. ALZpath p‐Tau217, Lilly p‐Tau217 and Lilly p‐Tau181 were found to be significantly associated with plaque (ρ=0.58, ρ=0.78, ρ=0.65; p<0.001) and tangle (ρ=0.26, ρ=0.51, ρ=0.37; p<0.05) load, when adjusting for age, sex and time of interval between blood sampling and death (Table 1). All the three biomarkers were also found to be significantly associated with plaque load (r=0.53, r=0.73 and 0.59; p<0.001), when adjusting for tangle load. However, only Lilly p‐Tau217 was significantly associated with tau tangle load (r=0.32, p=0.007), when adjusting for plaques. The correlations of ALZpath p‐Tau217 and Lilly p‐Tau181 with plaque load were comparable but Lilly p‐Tau217 exhibited significantly higher correlations with plaques (ρ_diff_ =0.20; p=0.015) and tangles (ρ_diff_ =0.29; p=0.003) than ALZpath p‐Tau217. ALZpath p‐Tau217 and Lilly p‐Tau181 predicted the elevated levels of ADNC, tangle, and amyloid pathology with similar accuracy (AUCrange, 0.74‐0.79) but the AUC´s of Lilly p‐Tau217 were significantly higher in comparison to ALZpath p‐Tau217 (ADNC, AUC_diff_=0.12, p_diff_=0.021; Braak, AUC_diff_=0.08, p_diff_=0.021; CERAD, AUC_diff_=0.11, p_diff_=0.024) (Figure 1). When looking at the changes in biomarker levels for non‐AD pathologies, we found that only Lilly p‐Tau217 was significantly higher in participants with both AD and cerebral amyloid angiopathy (CAA) pathology in comparison to those with only AD and CAA pathology (Figure 2).

**Conclusion:**

ALZpath p‐Tau217 exhibited similar performance as Lilly p‐Tau181, but in this cohort associations between ALZpath p‐Tau217 and the core measures of AD pathology were significantly lower in comparison with Lilly p‐tau217. These findings will need to be replicated in larger independent cohorts.